# Supplementary material for: Structural basis for UFM1 transfer from UBA5 to UFC1
Source: Nat Commun. 2021 Sep 29;12:5708. doi: 10.1038/s41467-021-25994-6 (PMC8481289; doi:10.1038/s41467-021-25994-6)
Supplement: Supplementary file 1 — Supplementary info [file 41467_2021_25994_MOESM1_ESM.pdf]

Supplementary information

Structural basis for UFM1 transfer from UBA5 to UFC1

Manoj Kumar [1], Prasanth Padala[1†], Jamal Fahoum[1], Fouad Hassouna[1], Tomer Tsaban[2] Guy Zoltsman[3], Sayanika Banerjee[1], Einav Cohen-Kfir[1], Moshe Dessau[4], Rina Rosenzweig[3], Michail N. Isupov[5], Ora Schueler-Furman[2] and Reuven Wiener[1*]

[1]Department of Biochemistry and Molecular Biology, The Institute for Medical Research Israel-Canada, Hebrew University-Hadassah Medical School, Jerusalem 91120, Israel

[2]Department of Microbiology and Molecular Genetics, Institute for Medical Research Israel-Canada, Faculty of Medicine, the Hebrew University of Jerusalem 91120, Israel

[3]Department of Structural Biology, Weizmann Institute of Sciences, Rehovot, Israel

[4]Azrieli Faculty of Medicine, Bar-Ilan University, Safed 1311502, Israel.

[5]The Henry Wellcome Building for Biocatalysis, Biosciences, University of Exeter, Stocker Road, Exeter, EX4 4QD, United Kingdom

[†]Present address: Department of Biochemistry, School of Biomedical Sciences, University of Otago, Dunedin 9054, New Zealand

[*]Corresponding author: Reuven Wiener (reuvenw@ekmd.huji.ac.il)

Supplementary information Inventory:

Supplementary Tables 1-4

Supplementary Figures 1-9

Supplementary Table 1: Data collection and Refinement statistics of crystal structures

Structure	UFC1-UBA5 (389-404)	UBA5 (347-404)-UFC`1	UFC1 Y110A&F121A
Beamline	ESRF ID23	BESSY II BL14.1	XtalLab Pro (Rigaku) Pilatus 200K detector (Dectris)
Wavelength (Å)	0.9720	0.9763	1.54
Space group	$P2_12_12_1$	$P6_4$	$P\ 4_12_12$
Unit Cell a, b, c (Å), α, β, γ (°)	47.1, 67.2, 133.8, 90, 90, 90	84.6, 84.6, 60.8, 90, 90, 120	46.16 46.16 143.76 90 90 90
Resolution range (Å)[a]	33.71 – 2.40 [b] (2.47-2.40) [b]	46.78-2.65 (2.78-2.65)	33.24 - 2.20 (2.28 - 2.20)
Total reflections [a]	297,196 (14,591) [b]	149,370 (20,269)	10,416 (917)
Unique reflections [a]	14,084 (704) [b]	7,314 (953)	8,488 (804)
Completeness sperical (%)[a]	81.3 (49.4) [b]	100.0 (100.0)	99.82 (99.14)
Multiplicity [a]	21.1 (20.7)	20.4 (21.4)	1.2 (1.1)
R_{meas} (%) [a,c]	13.6 (142.9) [b]	11.2 (600.2)	15.3 (71.9)
$<I>/<\sigma(I)>$ [a]	16.9 (2.5) [b]	13.1 (0.6)	13.3 (2.2)
$CC_{1/2}$ [a,d]	0.999 (0.868) [b]	0.990 (0.292)	0.99 (0.47)
Wilson B-factor [e] (Å^2)	52.1 [b]	127.1	25.44
Refinement statistics			
R_{work}	0.219	0.219	0.205
R_{free}	0.288	0.264	0.256
No. of protein monomers in a.u	2	1	1
Number of atoms			
Macromolecules	3,000	1,488	1,316
Solvent	52	-	59
Number of protein residues	354	183	164
RMS bond lengths (Å)	0.007	0.004	0.013
RMS bond angles (°)	1.59	1.45	1.82
Ramachandran favored (%)[f]	95.7	86.7	97.5
Ramachandran allowed (%)	4	13.3	2.5
Ramachandran outliers (%)[f]	0.3	0.0	0.0
Clashscore [f]	8.9	21.2	10.23
Average B-factor protein (Å^2)	60.1	157.1	28.77
Average B-factor solvent (Å^2)	40.6	-	33.38

[a]Values for the highest resolution shell are given in parentheses

[b] Values are given for data subjected to anisotropic ellipsoidal truncation using the STARANISO server[1]

[c] $R_{meas} = \Sigma_h[m/(m - 1)]^{1/2} \Sigma_i|I_{h,i} - <I_h>|/ \Sigma_h \Sigma_i I_{h,i}$

[d] $CC_{1/2}$ is defined in [2]

[e] Wilson B-factor was estimated by SFCHECK[3].

[f] The Ramachandran statistics and clashscore statistics were calculated using MOLPROBITY.

Supplementary Table 2: Thermodynamic parameters of binding experiments measured by isothermal titration calorimetry

Experiment	[Syr] (M)	[Cell] (M)	N1 (sites)	N1 Error	N1 Dependency	KD1 (M)	KD1 Error (M)	KD1 Dependency	N2 (sites)	N2 Error	N2 Dependency	KD2 (M)	KD2 Error (M)	KD2 Dependency
**UBA5 (Δ349-389) into UFC1**	1.50E-03	1.50E-04	1.07	0.131	0.995	5.50E-06	8.69E-07	0.887	6.80E-03	0.139	1	8.00E-07	7.55E-07	0.996
**UBA5 (333-404) into UFC1**	1.60E-03	1.60E-04	1.26	4.6E-02	0.989	2.54E-06	8.67E-08	0.659	1.4E-02	4.6E-02	0.996	5.64E-08	3.98E-08	0.601
**UBA5 (Y372A) into UFC1**	8.7E-04	9E-05	6.8E-02	8.9E-02	0.997	1.22E-07	1.02E-07	0.969	1.11	8.3E-02	0.992	1.94E-06	1.57E-07	0.805
**UBA5 1-404 M401R into UFC1**	9.00E-04	1.00E-04	N/A	N/A	N/A	N/A	N/A	N/A	N/A	N/A	N/A	N/A	N/A	N/A
**UBA5 1-404 L397R into UFC1**	9.00E-04	1.00E-04	N/A	N/A	N/A	N/A	N/A	N/A	N/A	N/A	N/A	N/A	N/A	N/A

Supplementary Table 3: predicted pKa and buried surface area (BSA) of the active site cysteine in E2 proteins

**Protein**	**PDB code**	**Catalytic Cysteine**	**BSA**	**pKa**
UFC1	2Z6P	116	4%	8.51
UBC9	1U9A	93	36%	11.28
UBCH5C	1X23	85	26%	11.33
UBC12	1Y8X	111	31%	10.91
UBCH7	4Q5H	86	18%	9.57
UBCH5B	3TGD	85	23%	11.13
UBC13	1JBB	87	16%	9.86
UBCH5A	2C4P	85	37%	10.79
UBCH10	4YII	114	17%	9.13
UBE2K	1YLA	92	24%	10.27
UBCH6	5LBN	131	21%	10.25
RAD6B	2YBF	88	23%	9.25
UBE2F	3FN1	116	37%	9.49
UBC7	2CYX	89	49%	9.47
UBE2H	2Z5D	106	19%	9.02
UBE2S	5BNB	95	30%	9.28
UBE2T	1YH2	86	31%	9.77
UBE2U	1YRV	89	31%	10.44
E2E2	1Y6L	85	22%	9.56
UBCH8	1WZV	85	18%	9.63
UBC1	2AAK	88	15%	9.47
RAD6A	6CYO	88	22%	9.86
CDC34	3RZ3	93	31%	10.01
ATG3	2DYT	234	35%	10.29

Supplementary Table 4: Cavity volume in selected E2 proteins

**Protein**	**Volume (Å^3)**	**Residues defining the cavity**
UFC1-2Z6P	377	T93, A94, P95, E96, I97, Y110, K114, I115, C116, L117, T118, D119, F121, K122, L137, G141, L142
UFC1 Y110A and F121A	126	P95, E96, I97, I115, I117, K122, L137
UBC13-1JBB	163.1	P67, K68, V69, R85, I86, L91, K92
UBC12-1Y8X	110.4	P91, K92, V93, K94, V110, L115, R116
UBC9-1U9A	No proper cavity; 16.6	V92, C93, L94, L97, E98
UBCH5B-3TGD	No cavity	
UBCH57-4Q5H	66.1	K67, I68, Q84, V85
UBCH5C-1X23	No cavity	
E2E2-1Y6L	No cavity	
UBCH8-1WZV	No cavity	
UBC1-2AAK	No cavity	
RAD6A-6CYO	No proper cavity: 5.0	P68, T69, L92, Q93
UBCH5A-2C4P	No cavity	
UBCH10-4YII	No cavity	
UBE2K-1YLA	No cavity	
UBCH6-5LBN	No cavity	
RAD6B-2YBF	163.8	K66, P67, P68, T69, V70, R71, S86, I87, L92, Q93
UBE2F-3FN1	89.1	P95, P96, K97, V98, E114, I115, L120
UBC7-2CYX	96.3	P69, K70, M71, Y83, R87, V88, C89, L93, H94
UBE2H-2Z5D	98.2	R73, D75, S86, I87, G88, M90, S102, T104
UBE2S-5BNB	198.1	L64, P74, P75, K76, G77, E93, I94, C95, V96, L99, K100, V112, I116
UBE2T-1YH2	No cavity	
UBE2U-1YRV	82.8	P68, V69, V70, Q87, P88, L93, D94
CDC34-3RZ3	No proper cavity: 6.8	V92, I94, L97

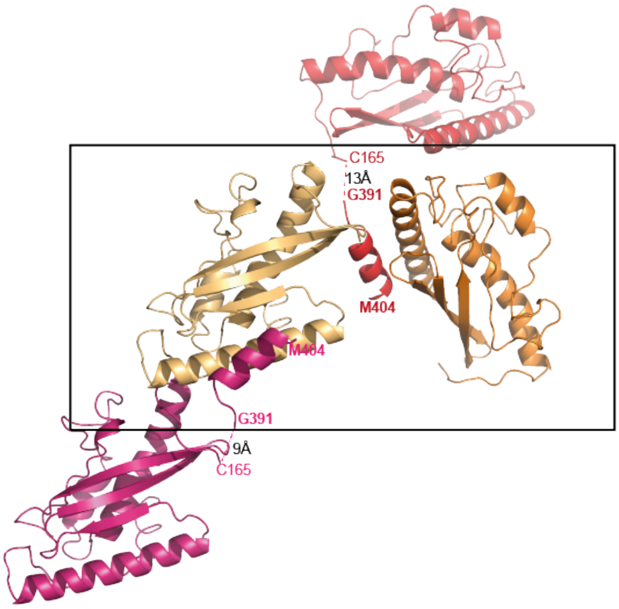

Supplementary Figure 1: The composition of the UFC1-UBS asymmetric unit. The asymmetric unit (black rectangle) comprises two molecules of UFC1 (orange and light orange) and two molecules of UBS (red and magenta). The UBS molecules shown in the asymmetric unit are fused to UFC1 molecules that arrive from other asymmetric units. The first two residues of UBS (D389 and S390) and the last two residues of UFC1 (N166 and Q167) were not modeled due to the absence of electron density. The first and last seen residues of UBS are shown in bold while the last seen residues of UFC1 is shown in regular font. The first and last seen residues of UBS and UFC1, respectively, are connected by dotted line and the distance shown could be spanned by the missing residues.

A

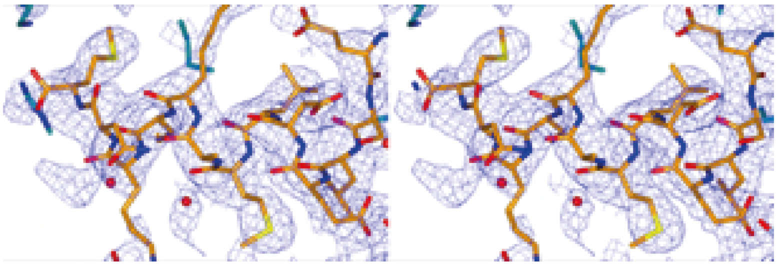

B

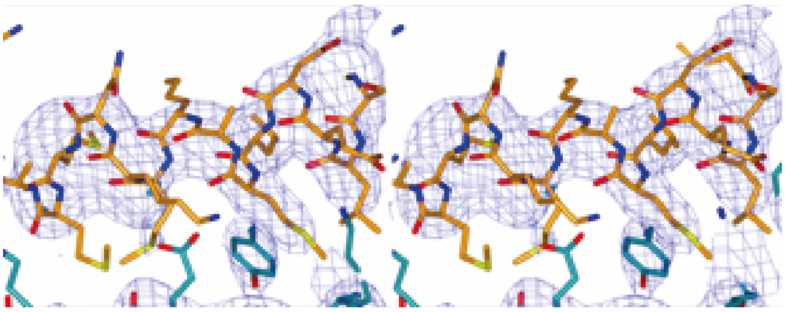

Supplementary Figure 2: Stereographic representations of the UBS helical region with a composite OMIT electron density map contoured at 1 σ. The map coefficients were calculated using Phenix[4] and the figure was prepared using CCP4mg software[5]. **A.** In the UFC1-UBS structure, the UBS region and its partner UFC1 region are shown as stick models with carbon atoms in orange and cyan, respectively. **B.** In the UBA5(347-404)-UFC1 structure the UBS region and the binding region of the crystallographically related UFC1 are shown as stick models with carbon atoms in orange and cyan, respectively.

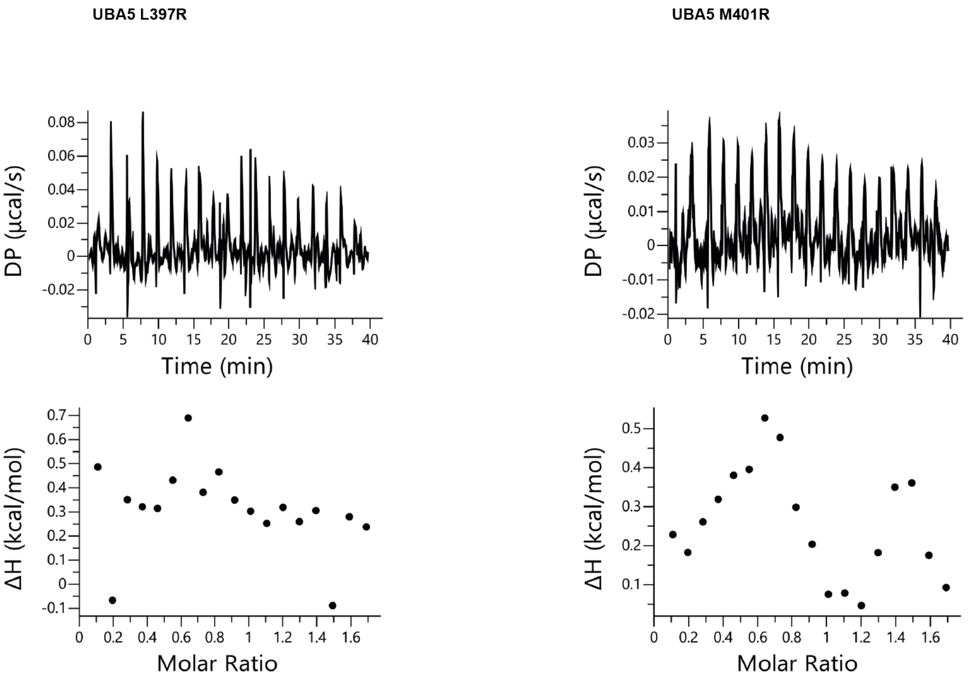

Supplementary Figure 3: Mutations in UBS prevent binding to UFC1. ITC experiment showing no binding affinity between UFC1 and indicated UBA5 mutations. Top graph represents raw data of heat flow versus time. The area under the peaks of the upper panel was integrated and plotted as kcal per mole of UFC1 as a function of binding stoichiometry in the bottom panel. Graph represents the enthalpy change versus the molar ratio of UFC1 to UBA5 mutants. Both graphs show no binding of UFC1 to the above mutants.

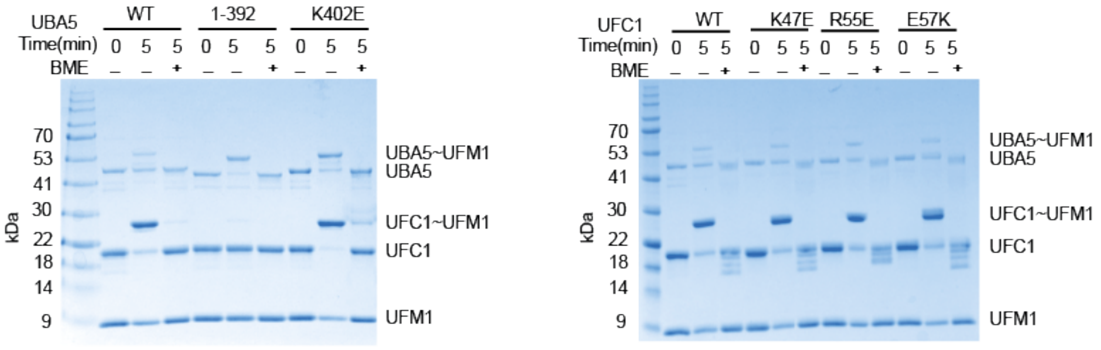

Supplementary Figure 4: Mutations in the indicated charged residues of UFC1 or UBS hardly affect UFM1 transfer. SDS-PAGE analysis showing the charging of UFC1 WT or mutant with WT or the indicated UBA5 mutants. The gels are representative of two independent experiments. Source data are provided as a source data file.

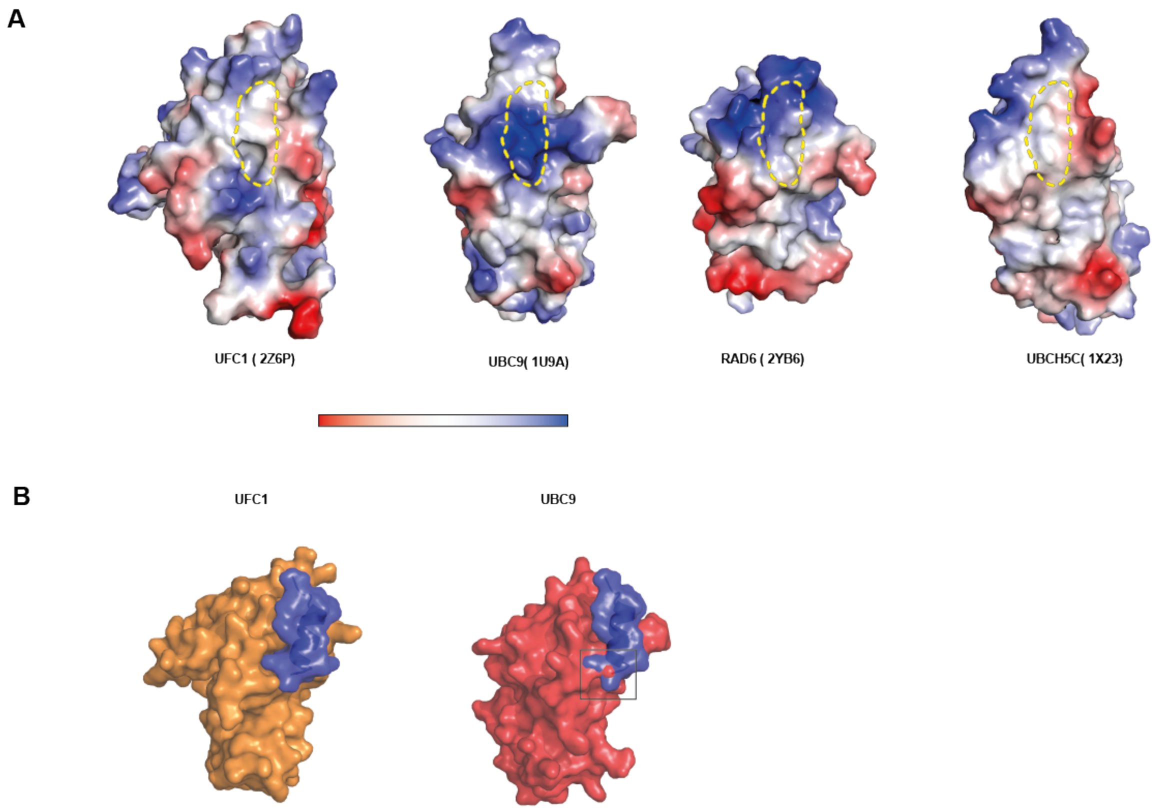

Supplementary Figure 5: The surface for UBS binding is unique to UFC1. **A.** A comparison between electrostatic potential surfaces of indicated E2 enzymes. Yellow dotted line corresponds to the border of UFC1 surface that binds the UBS and the corresponding surface in other E2 proteins. B. Clashes of UBS (blue) with UBC9 (red) surface (indicated with black box) but not with UFC1(orange) surface.

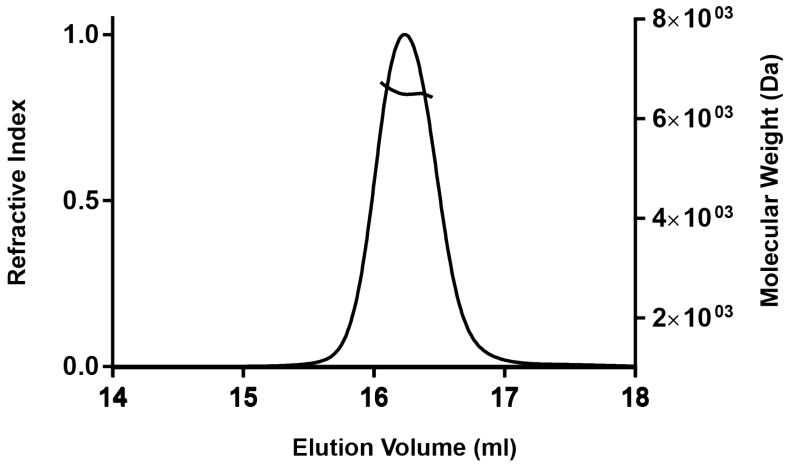

Supplementary Figure 6: Size exclusion chromatography with multi-angle light scattering analysis of UBA5 (347-404) fragment. Protein was loaded on an analytical SEC column (Superdex 75 10/300 GL) equilibrated with a buffer containing 20 mM Tris (pH 7.5), 50 mM NaCl. Molecular mass within the chromatographic peak was calculated using ASTRA software, version 7 (Wyatt Technologies). The measured molecular weight of 6.6 kDa fits the expected molecular weight of this fragment (6.4 kDa). Source data are provided as a source data file.

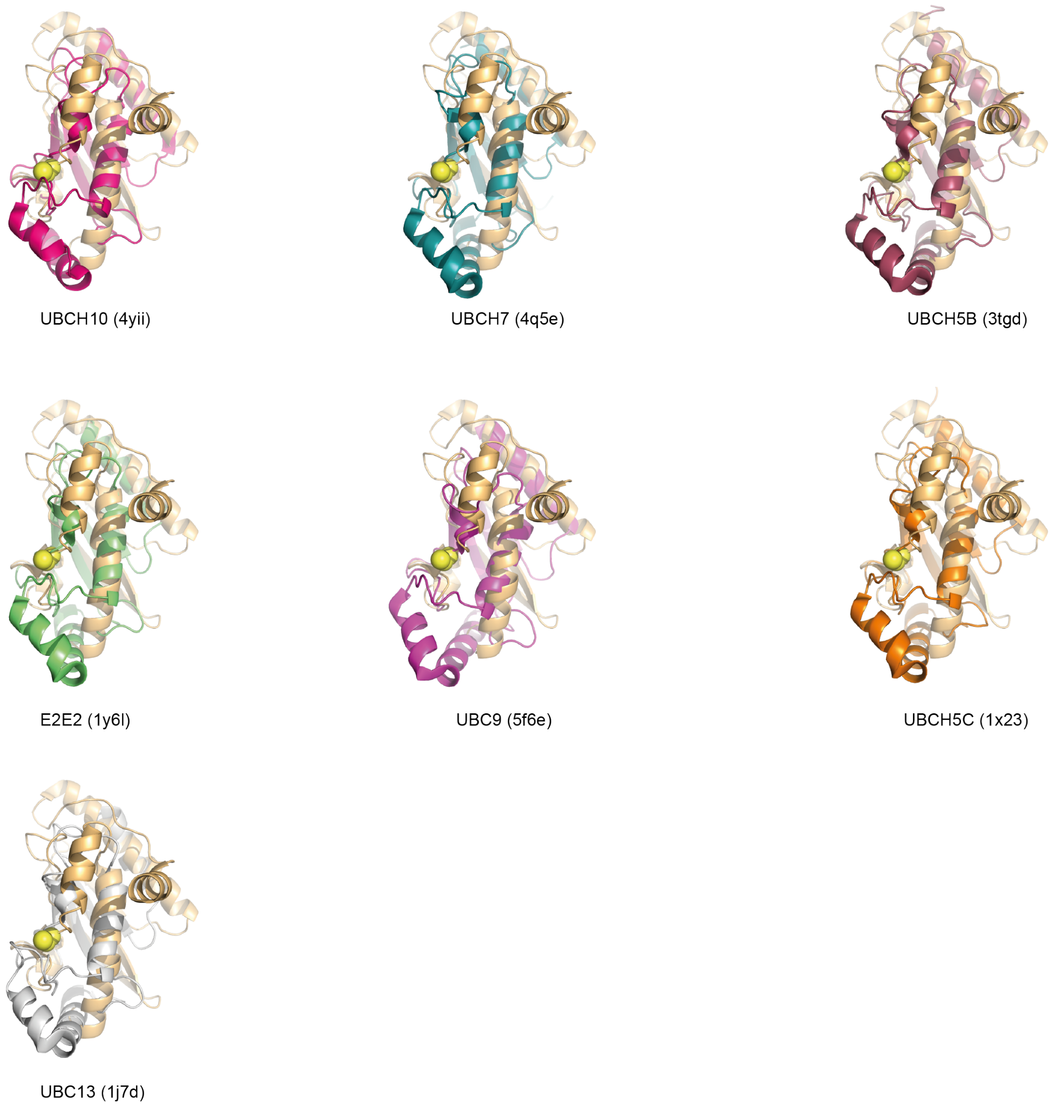

Supplementary Figure 7: Superpositions of UFC1 with indicated E2 enzymes. The active site Cys is in yellow sphere. UFC1 is colored as in Fig.1C.

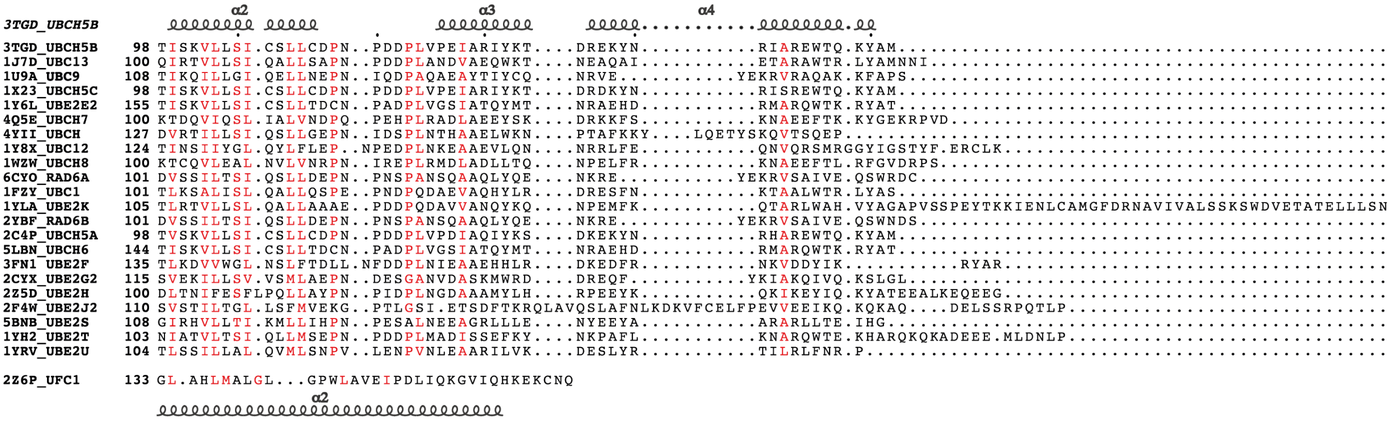

Supplementary Figure 8: sequence alignment of E2 enzymes. The secondary structural elements covering the region of helix 2 to the C-terminus are shown for the indicated E2s (top). The corresponding secondary element for that region in UFC1 is shown underneath the sequence alignment.

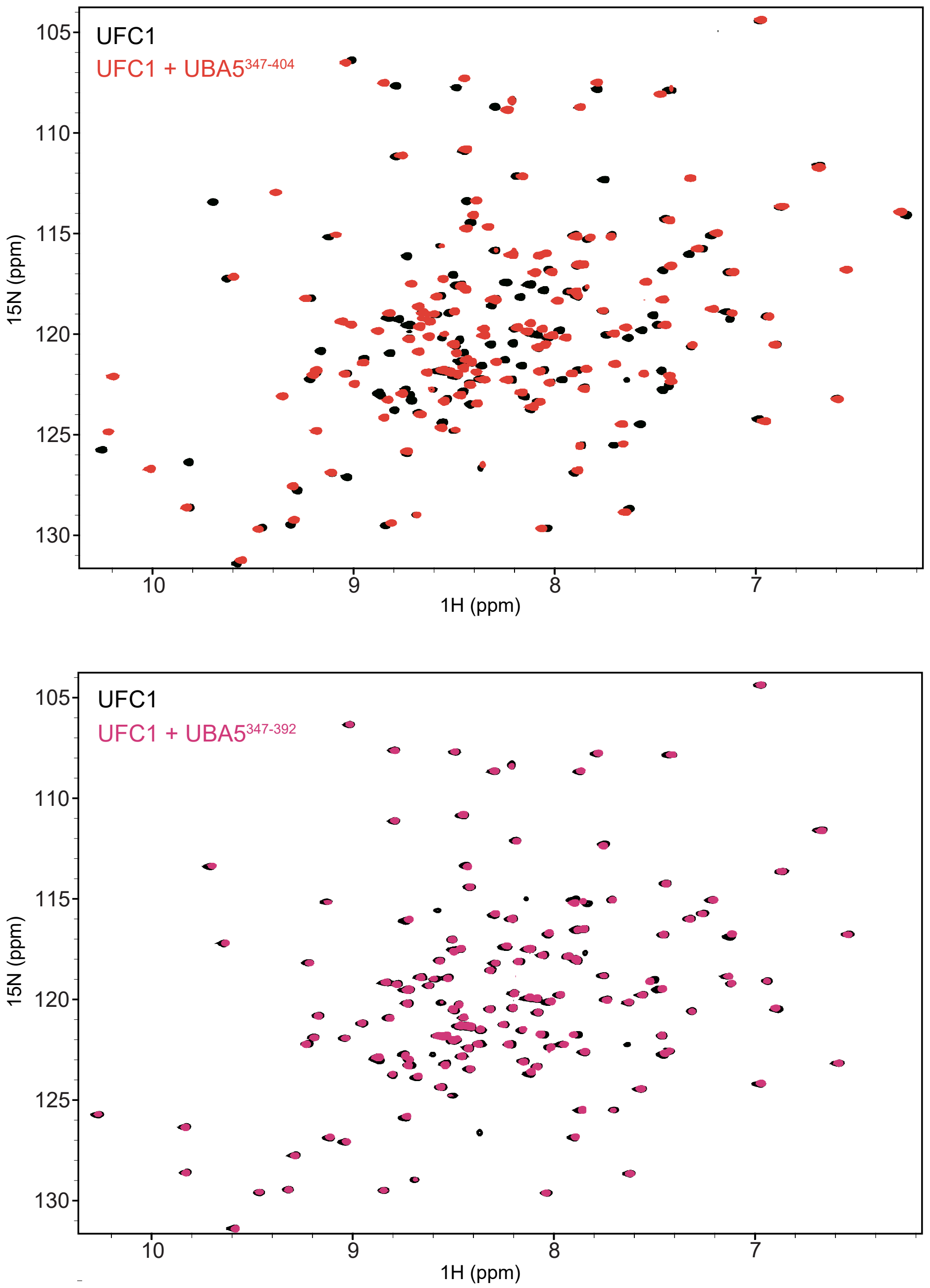

Supplementary Figure 9: Overlay of ^{1}H–^{15}N HSQC spectra of 0.2 mM UFC1 alone(black), and in the presence of equimolar concentration of UBA5$^{347\text{-}404}$ (red, upper panel) or five-fold excess of UBA5$^{347\text{-}392}$ (pink, bottom panel).

Supplementary References

1 Tickle, I. Staraniso: Use of a Webgl-Based 3-D Interactive Graphical Display to Represent and Visualise Data Quality Metrics for Anisotropic Macromolecular Diffraction Data. *Acta Crystallogr A* **75**, E162-E162, doi:10.1107/S205327331909394x (2019).

2 Karplus, P. A. & Diederichs, K. Linking Crystallographic Model and Data Quality. *Science* **336**, 1030-1033, doi:10.1126/science.1218231 (2012).

3 Vaguine, A. A., Richelle, J. & Wodak, S. J. SFCHECK: a unified set of procedures for evaluating the quality of macromolecular structure-factor data and their agreement with the atomic model. *Acta Crystallogr D* **55**, 191-205, doi:Doi 10.1107/S0907444998006684 (1999).

4 Afonine, P. V. *et al.* Towards automated crystallographic structure refinement with phenix.refine. *Acta crystallographica. Section D, Biological crystallography* **68**, 352-367, doi:10.1107/S0907444912001308 (2012).

5 Potterton, L. *et al.* Developments in the CCP4 molecular-graphics project. *Acta crystallographica. Section D, Biological crystallography* **60**, 2288-2294, doi:10.1107/S0907444904023716 (2004).
