# Supplementary material for: Structural basis for UFM1 transfer from UBA5 to UFC1
Source: Nat Commun. 2021 Sep 29;12:5708. doi: 10.1038/s41467-021-25994-6 (PMC8481289; doi:10.1038/s41467-021-25994-6)
Supplement: Supplementary file 3 — Description of Additional Supplementary Files [file 41467_2021_25994_MOESM3_ESM.docx]

**Description of Additional Supplementary Files**

**Supplementary Data 1:**

Atomic coordinates of the UFC1/UBA5-fragment docking model with UBA5 residue Y372 closely stacked against UFC1 Y110.

**Supplementary Data 2:**

Atomic coordinates of the UFC1/UBA5-fragment docking model with UBA5 residue Y372 pointing towards UFC1 F121.

**Supplementary Data 3:**

Atomic coordinates of the ternary UBA5/UFM1/UFC1 docking model.
